# Antibiotic Resistance and Virulence Factors in Clinical Isolates of *Stenotrophomonas maltophilia* from Hospitalized Patients in Tehran, Iran

**DOI:** 10.1155/2024/8224242

**Published:** 2024-10-01

**Authors:** Mahrokh Bahrami, Narjess Bostanghadiri, Mehdi Goudarzi, Niloufar Khodaei, Ali Hashemi

**Affiliations:** ^1^ Department of Microbiology School of Medicine Shahid Beheshti University of Medical Sciences, Tehran, Iran; ^2^ Department of Microbiology School of Medicine Iran University of Medical Sciences, Tehran, Iran

## Abstract

*Stenotrophomonas maltophilia* causes challenging infections in immunocompromised patients, exhibiting increasing resistance to multiple antimicrobials and possessing various virulence genes, including emerging resistance to trimethoprim-sulfamethoxazole. A total of 80 clinical isolates of *S. maltophilia* were collected from multiple hospitals in Tehran, Iran. This study conducted an analysis of antibiotic susceptibility by disc diffusion method and *E*-test assay, resistance and virulence gene frequencies were examined by PCR-sequencing, and multilocus sequencing typing (MLST) was performed for strain typing. Across the tested isolates, we observed notably high resistance rates for imipenem 80 (100%), meropenem 78(97.5%), and ceftazidime 72 (90%), while trimethoprim-sulfamethoxazole (SXT) showed a lower resistance rate of 2 (2.5%). Minocycline and levofloxacin demonstrated the highest susceptibility rates, with 70 (87.5%) and 80 (100%), respectively. The prevalence of antibiotic resistance genes bla_*L*1_, and bla_*L*2_ was 71 (88.75%) and 76 (95%), respectively. Additionally, the PCR analysis revealed that the frequency of virulence genes (*fliC*, *virB*, *papD*, *pilU*, *hlyIII*, *stmPr1*, and *stmPr2*) was 78 (97.5%), 77 (96.25%), 58 (72.5%), 77 (96.2%), 76 (95%), 31 (38.75%), and 80 (100%), respectively. Resistance to SXT isolate belong to the sequence type (ST15) and exhibits allelic profiles of (10, 29, 21, 21, 32, 32, and 10). The data obtained from our investigation have indicated that SXT remains an efficacious antibiotic and also highlighted the importance of effective management, identification of resistant isolates, and typing methods to address the global prevalence of antibiotic resistance in *S. maltophilia*.

## 1. Introduction

*Stenotrophomonas maltophilia* is a common Gram-negative bacterium known for its resistance to antibiotics. It has the potential to act as an opportunistic pathogen, particularly in vulnerable populations such as individuals undergoing chemotherapy for hemato-oncologic diseases [1–3]. It is pathogenic in individuals with malignancy, immunodeficiency, chronic respiratory diseases, or those taking long-term antibiotics [4, 5]. The recent surge in infections among ICU patients and newborns includes hospital-acquired infections, bloodstream infections, pneumonia, skin/soft tissue infections, catheter-related infections, urinary tract infections, surgical site infections, and endophthalmitis [6, 7]. The prevalence of *S. maltophilia* has been increasing during the COVID-19 pandemic, along with other pathogens such as *Acinetobacter baumannii* and *Mycobacterium tuberculosis* [8–10].

To understand *S. maltophilia*'s pathogenicity, a thorough genetic investigation is crucial. Key genetic components include the *FliC* gene, which produces the flagellum. The flagellum in bacteria is not just for movement; it is also essential in causing infections. It helps the bacteria stick to and invade host cells, form biofilms, and even start immune responses in the infected host [11, 12]. Additionally, *stmPr1* and *stmPr2*, the primary and secondary serine proteases, degrade immune cells and extracellular matrix proteins, causing human disease. *StmPr1*, notably, triggers harmful processes in human lung cells, potentially contributing significantly to how *S. maltophilia* causes illness. Although less is known about *stmPr2*, its connection to the bacterium's secretion system suggests that it might also play a role in causing disease [13]. Other critical genes include *hly*, related to hemolysin, a protein that breaks down red blood cells, *virB* involved in Type IV secretion systems and about 86.6% of *S. maltophilia* strains carry it, linked to a system used for delivering molecules to host cells, the *pilU* gene, present in 96.6% of strains, contributes to twitching motility powered by type IV pili, and *papD* associated with colonization [14–16].

*S. maltophilia* has developed resistance to antibiotics, and it was previously sensitive to including fluoroquinolones, aminoglycosides, and *β*-lactams [17, 18]. This resistance is due to several factors, such as structural changes in antibiotics, efflux pumps, reduced membrane permeability, and beta-lactamase production [19, 20]. The bacterium has two inducible beta-lactamases, bla_*L*1_ and bla_*L*2_, classified as B and A in the Ambler classification, respectively [17]. bla_*L*1_ (MBL) is responsible for resistance to beta-lactams other than monobactams, while bla_*L*2_ is a cephalosporinase that is sensitive to clavulanic acid and resistant to cephalosporins, penicillins, and aztreonam [17, 21, 22]. bla_*L*1_, an inherent chromosomal gene, and bla_*L*2_, identified through comprehensive genome analysis, actively contribute to the bacterium's natural resistance against *β*-lactams, including the vital carbapenems. This resistance mechanism undermines the effectiveness of these antibiotics, often considered a last line of defense against infections caused by multidrug-resistant bacteria [23, 24]. Trimethoprim-sulfamethoxazole (SXT) is often the first choice for treating *S. maltophilia* infections because it works well against this bacterium. MLST, a method used to explore genetic relationships in bacteria, helps researchers track how resistance genes, potentially including those resisting SXT, spread in *S. maltophilia* [25–27]. Further research and identification of alternative antibiotics are needed to address this issue.

*S. maltophilia* can be grouped into three Genovars based on 16SrRNA gene analysis, with two associated with environmental isolates and one with clinical and aquatic isolates [28]. The main DNA fingerprinting methods used for typing *S. maltophilia* strains are PCR-restriction fragment length polymorphism (PCR-RFLP), and amplified fragment length polymorphism (AFLP), with other available methods including pulsed-field gel electrophoresis (PFGE), *gyrB* RFLP, repetitive extragenic palindromic (rep)-PCR, DL automated rep-PCR, multiple-locus variable number of tandem repeats analysis (MLVA), and multilocus sequence typing (MLST) [29]. MLST is a high-resolution genetic technique that uses seven housekeeping genes as persistent markers for comparing strains over time and geography [30]. MLST is preferred over other typing methods for studying antibiotic resistance and virulence factors in clinical isolates of *S. maltophilia* due to its high resolution and reliability in understanding genetic diversity. It offers a comprehensive view of relationships between isolates, their resistance profiles, and virulence traits, making it valuable in epidemiological and clinical studies [29]. The antibiotics levofloxacin, minocycline, imipenem, meropenem, and SXT are chosen for studying *S. maltophilia* resistance due to their effectiveness in treating its infections. They help identify resistance patterns, guiding effective treatment strategies for this bacterium [31, 32].

The primary aim of the present investigation was to appraise the patterns of antimicrobial resistance and pathogenic factors' genes among the clinical *S. maltophilia* strains isolated from several hospitals in Tehran, Iran. Furthermore, the research also scrutinized the genetic diversity of the isolates.

## 2. Methods

### 2.1. Ethical Considerations

The Ethics Committee of Shahid Beheshti University of Medical Sciences (IR.SBMU.MSP.REC.1398.806) provided ethical approval for this study. To ensure participant privacy, no personal data were gathered or included in the research, and all participants remained anonymous.

### 2.2. Bacterial Species Isolation and Identification Process

This was a retrospective study. A total of 80 nonduplicate clinical isolates of *S. maltophilia* were obtained randomly from several hospitals in Tehran, Iran, from January 2018 to January 2019. The laboratory employed standard biochemical methods to identify the isolates. To confirm the *S. maltophilia* isolates, 16S rRNA gene amplification and sequencing techniques were employed (as indicated in Table 1) [34]. The isolates were stored in the Luria–Bertani (LB) liquid medium (Merck company-Germany) supplemented with 20% glycerol and maintained at −70°C. Additionally, quality control strains, including *Escherichia coli* ATCC 25922 and *Pseudomonas aeruginosa* ATCC 27853, were included in the study.

### 2.3. Antimicrobial Susceptibility Testing (AST)

The susceptibility of *S. maltophilia* isolates was assessed following the guidelines provided by the Clinical and Laboratory Standards Institute (CLSI) [35]. The Kirby–Bauer disc diffusion technique was employed to test the susceptibility to various antibiotics, including levofloxacin (5 *μ*g), minocycline (30 *μ*g), imipenem (10 *μ*g), meropenem (10 *μ*g), ceftazidime (30 *μ*g), and sulfamethoxazole/trimethoprim (1.25/23.75 *μ*g) (MAST Diagnostics, Merseyside, UK). The MIC-Test Strip (Liofilchem Italy) was utilized to measure the minimal inhibitory concentration (MIC) for two antibiotics, including SXT and ceftazidime. Muller–Hinton agar plates with disks or strips were incubated in ambient air at 35°C ± 2°C and read after 20 to 24 h of incubation. The bacteria's sensitivity or resistance was assessed using the values found in the CLSI guidelines. *Escherichia coli* ATCC 25922 and *Pseudomonas aeruginosa* ATCC 27853 were used as controls.

### 2.4. Polymerase Chain Reaction Sequencing Technique (PCR)

Genomic DNA was extracted by the High Pure PCR Template kit (GeNet Bio Company, Daejeon, Korea; Cat. No, *K*-3000) as per the manufacturer's guidelines and utilized for the detection of resistance genes (bla_*L*1_ and bla_*L*2_) and virulence genes (*virB*, *fliC*, *stmPr1*, *stmPr2*, *pilU*, *hlyIII*, and *papD*) through the Polymerase Chain Reaction (PCR) technique. PCR was performed in 25 *μ*l reaction volumes using specific primers listed in Table 1. The PCR reaction mixture included 1 *μ*l (20 ng) of DNA template, 1 × PCR buffer, 12.5 *μ*l of 2 × Master Mix (SinaClon-Iran), 3 mmol/L MgCl2, 0.4 mmol/L dNTPs, 9.5 *μ*l of sterile distilled water, 1 *μ*l of 10 pmol of each primer, and 0.08 IU of Taq DNA polymerase. The negative control in all analyses consisted of all the components of the reaction mixture, excluding bacterial genomic DNA. In addition, we used *S. maltophilia* strain ng 49 and strain as 40 with accession numbers MF458984 and MF497329, which were submitted in a previous study as bla_*L*1_ and bla_*L*2_ positive controls, respectively [30].

The PCR conditions comprised an initial denaturation at 94°C for 5 min, followed by 36 cycles of denaturation at 94°C for 45 s, annealing at 56–62°C (depending on the gene-specific primers) for 45 s, and extension at 72°C for 45 s. A final extension step was performed at 72°C for 5 min. The resulting PCR products were then analyzed using agarose gel electrophoresis (1% gel), stained with DNA Safe, and visualized under UV light. For sequencing and verification, the PCR products were sent to Shahid Rajaee Hospital in Tehran, Iran. The obtained sequences were further analyzed using MEGA-4 and Chromas 1.45 software and confirmed through Blast on the NCBI website.

### 2.5. Multilocus Sequence Typing (MLST)

The MLST technique was implemented to analyze the molecular typing of *S. maltophilia* isolates which were resistant to SXT [36]. The entire putative coding sequences of the housekeeping genes were selected from the PubMLST website (https://pubmlst.org/organisms/stenotrophomonas-maltophilia/) (Table 1). A reaction volume of 25 *μ*l was prepared for the Polymerase Chain Reaction (PCR) using specific primers. The components included 3 *μ*l (20 ng) of the DNA template, 12.5 *μ*l of 2 × Master Mix (SinaClon-Iran, CAT. No., PR901638), 1 × PCR buffer, 0.4 mmol/L deoxy-nucleotide triphosphates (dNTPs), 3 mmol/L MgCl2, 0.08 IU Taq DNA polymerase, 1 *μ*l of 10 pmol of each primer, and 7.5 *μ*l of sterile distilled water. The PCR products were analyzed on an agarose gel and were then subjected to sequencing by Rajaie Hospital, Tehran, Iran. The sequence was analyzed using the Chromas version 1.45 software. In addition, sequence alignment was conducted by the Nucleotide BLAST program.

The obtained sequences of each seven genes submitted to the PUBMLST.ORG website to determine the allele number and specific sequence type (ST) [33]. A combination of the allelic sequences of the seven genes yielded the allelic profile for each isolate.

### 2.6. Statistical Analysis

SPSS v.20.0 software (SPSS Inc. Chicago, IL, USA) and the chi-squared test were used for the statistical analyses. In this study, statistical significance was defined as a *p* value of less than 0.05.

## 3. Results

### 3.1. Patient Background and Bacterial Isolation

In the present cross-sectional study, 80 isolates of nonrepetitive *S. maltophilia* were gathered from hospitalized patients across various wards in four hospitals: Loghman-e Hakim Hospital (35 isolates), Children's Medical Center (30 isolates), Shariati Hospital (8 isolates), and Rasoul Akram Hospital (7 isolates). The majority of the *S. maltophilia* isolates (85%) were obtained from the bloodstream infection, with 11.25% from tracheal tubes, and 3.75% from pharyngeal secretions. Of the 80 isolates, 47 (58.75%) were from males and 33 (41.25%) were from females (male:female ratio = 1.25). The age of the patients ranged in age from 6 months to 80 years.

### 3.2. Antibiotic-Susceptibility Testing

Based on the interpretive criteria established by CLSI, approximately 80(100%), 78(97.5%), and 72(90%) of the isolates were resistant to imipenem, meropenem, and ceftazidime. It was observed that 2 (2.5%) isolates exhibited resistance towards SXT. The greatest sensitivity was seen in levofloxacin, SXT, and minocycline at 80 (100%), 75 (93.75%), and 70 (87.5%), respectively.  Table 2 displays the percentage of isolates demonstrating resistance, intermediate, or susceptibility to the antimicrobial agents and along with the MIC ranges, MIC50 and MIC90.

**Figure 1:**
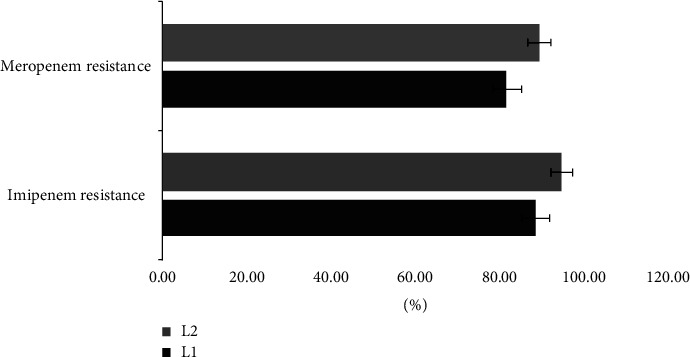
Prevalence of antibiotic resistance genes (bla_*L*1_ and bla_*L*2_) among imipenem and meropenem resistance isolates (*p* ≤ 0.001).

PCR-based resistance genes revealed that from 78 isolates resistant to meropenem, 82% had bla_*L*1_ and 90% had bla_*L*2_. Among 80 isolates where resistance to imipenem 88.75% and 95% carrying bla_*L*1_ and bla_*L*2_, respectively (Figure 1).

### 3.3. Virulence Gene Detection

The results of the virulence gene detection showed that the positive rates of the seven virulence genes were 78/80 (97.5%) for *fliC*, 77/80 (96.25%) for *virB*, 58/80 (72.5%) for *papD*, 77/80 (96.2%) for *pilU*, 76/80 (95%) for *hlyIII*, 31/80 (38.75%) for *stmPr1*, and 80/80 (100%) for *stmPr2*. There were 31 isolates of *S. maltophilia* that carried all four of the genes (Table 3). The statistical results showed no significant (*p* > 0.05) association between the presence of virulence genes and antibiotic resistance.

### 3.4. Multilocus Sequence Typing (MLST) Analysis

Two strains with resistance to SXT underwent molecular typing. As presented in Table 4, one of the strains belonged to ST15 based on MLST analysis and exhibited allelic profiles of (10, 29, 21, 21, 32, 32, and 10). The other strain's identity remained undetermined.

## 4. Discussion

*S*. *maltophilia* is a Gram-negative bacterium that has been identified as an opportunistic pathogen, particularly in patients with weakened or impaired immune systems. This organism has been linked to the development of various medical conditions such as meningitis, pneumonia, bacteremia, urinary tract infections, and respiratory issues in those suffering from cystic fibrosis [37]. The management of *S. maltophilia* poses significant challenges, owing to its inherent resistance to several antibiotics, and its ability to acquire novel resistance mechanisms via horizontal gene transfer and mutations [38].

In many countries, the rise of carbapenem resistance is alarming. Madrid, Spain, for instance, faces a critical situation with specific resistance rates of 99% for imipenem and 87.9% for meropenem. Moreover, in another country, all isolates demonstrated resistance to imipenem [39]. The ongoing investigation supports these findings, demonstrating significant carbapenem resistance with meropenem 78 (97.5%) and complete resistance with imipenem 80 (100%). Resistance towards carbapenems in *S. maltophilia* is attributed to a variety of mechanisms, which may include the expression of intrinsic *β*-lactamase. The present study has identified that a significant proportion of isolates (71 (88.75%) and 76 (95%) for bla_*L*1_ and bla_*L*2_ genes, respectively) harbor these genes. Notably, both genes were found in resistant to imipenem and meropenem samples, except for the higher amount of bla_*L*2_ compared to bla_*L*1_. In a study in China involving 118 *S. maltophilia* isolates, the positive rates for bla_*L*1_ and bla_*L*2_ genes were 75% (89/118) and 22% (26/118), respectively, and all 118 isolates exhibited resistance to carbapenems [40]. Additionally, an Iranian study reported that among 23 isolated *S. maltophilia* strains, 82.66% were beta-lactamase positive, and 60.86% were Metallo-beta-lactamase positive, with 39.13% of MBL-positive isolates also positive for the bla_*L*1_ gene [41]. These collective findings strongly suggest an association between the bla_*L*1_ and bla_*L*2_ genes and carbapenem resistance, corroborating our study's observations.

Our study's outcomes highlight a susceptibility rate of *S. maltophilia* isolates to ceftazidime, measured at 2.5%. These findings align with prior research, displaying MIC50 and MIC90 values of 8 and 32 *μ*g/ml, respectively [42]. Conversely, Jamali et al. and Ebrahim-Saraie reported a significantly higher susceptibility rate of 82%, 93.2% for ceftazidime, respectively [43, 44]. Moreover, variations in susceptibility rates for this drug were evident across regions: Latin America recorded rates of 93.3%, whereas the United States, Canada, and Turkey reported rates of 64.7%, 27%, and 67%, respectively [45]. Additionally, in a separate study conducted in China, the susceptibility to ceftazidime was lower at 20.0% [46], further emphasizing the variability in susceptibility observed across different geographical locations and research studies.

The recommended treatment for *S. maltophilia* involves SXT as the primary choice [47]. In our study, the MIC50 and MIC90 for this antibiotic were reported as ≤2.38 *µ*g/ml and ≤4.76 *µ*g/ml, respectively. Out of the 80 isolates tested, only 2 (2.5%) showed resistance, 3 (3.75%) were intermediate, and the majority, 75 (93.75%), were sensitive to SXT. In contrast, other studies have reported varying rates of resistance, ranging from 17.7% to 38.7% [21, 25, 48, 49]. In the recent meta-analysis, global resistance to this antibiotic was 14.6%, with the highest resistance rates reported in Asia (19.29%), Europe (10.52%), and the Americas (7.01%) [50, 51]. Our study observed a lower percentage of resistance to SXT compared to these reports. Insights from studies conducted by Jamali et al. and Madi et al. highlighted the sensitivity of *S. maltophilia* to SXT. Specifically, their findings showed MIC50 values of 0.5 *μ*g/ml and ≥4 *µ*g/ml and MIC90 values of 2 *μ*g/ml and ≥32 *µ*g/ml, respectively [52, 53]. Additionally, in our study, levofloxacin displayed complete sensitivity 80 (100%), while minocycline exhibited a high sensitivity rate of 70(87.5%). These findings are consistent with another study, reporting a 6.1% resistance rate for levofloxacin and complete sensitivity to minocycline [49]. Our research aligns with this by identifying minocycline as the most effective antibiotic. However, unlike the previous study, none of our isolates showed any resistance to levofloxacin, indicating a discrepancy in resistance percentages that might be attributed to differences in sample sizes. Another study by Madi et al. showcased a complete susceptibility of all isolated strains to levofloxacin [53]. Differences in *S. maltophilia* studies' findings may be due to various factors, namely, geographical variations in antibiotic use and local isolates, methodological differences in testing, time-related shifts in resistance patterns, sample size, and strain variations influencing antibiotic susceptibility. This disparity highlights the complexity and potential influencing factors in determining antibiotic resistance patterns across studies.

To gain a comprehensive understanding of the virulence of *S. maltophilia*, the isolates were subjected to thorough characterization based on the occurrence of specific potential virulence factors. *StmPr1* and *stmPr2* are extracellular serine proteases in *S. maltophilia*, breaking down proteins outside the cell. They impact antibiotic resistance alongside other mechanisms, influencing the bacterium's resistance profile [13, 44]. The frequency of *stmpr1* and *stmpr2* virulence genes was determined by Duan et al. to be 79.6% and 91.4%, respectively [54]. In contrast, among 80 isolates our study found the presence of the s*tmPr1* gene to be 31 (38.75%) and *Stmpr2* to be 80 (100%). According to a singular study, *stmPr1*, as opposed to *stmPr2*, may be a pertinent virulence factor of *S. maltophilia* [55]. This discrepancy may be a result of the more diverse isolates used in the previous study. Insufficient knowledge exists regarding the pathogenic mechanisms employed by *S. maltophilia*. Therefore, further investigation is required in this realm. In our study, among isolated, most of them had other genes which are related to the virulence factor in *S. maltophilia* and *fliC, virB, papD, pilU,* and *hlyIII* were 78 (97.5%), 77 (96.25%), 58 (72.5%), 77 (96.2%), and 76 (95%), respectively. Zamboni et al. found that the frequencies of *fliC*, *virB*, *papD*, *pilU*, *hlyIII*, and *stmPr1* virulence genes were 90%, 86.6%, 80%, 96.6%, 90%, and 50%, respectively [56]. These results are similar to those of our study. Saleh et al. reported the incidence of virulence genes, which comprised *fliC*, *stmPr1*, *virB*, *pilU*, *hlyIII*, and *papD*, among the isolates. The study revealed that 93%, 87%, 73%, 62%, 59%, and 57% of the isolates harbored these respective genes [57]. Except for *fliC*, there were discrepancies in the frequency of the other genes, which could explain the considerable difference in the number and type of isolates. These discrepancies highlight the need for further investigation into the pathogenic mechanisms employed by *S. maltophilia*. Understanding the prevalence and role of these virulence factors is crucial for developing effective treatment strategies against this pathogen.

The MLST method, based on the identification of 7 housekeeping genes of 500−450 bp in size, effectively compares strains across different times and geographical locations. This technique is a reliable indicator for distinguishing between intraspecies and interspecies *S. maltophilia* strains [58]. Distinct sequence types may carry varying sets of virulence genes, impacting the bacteria's pathogenicity, antibiotic resistance, and overall behavior in a host or environment [59]. In this study, trimethoprim-sulfamethoxazole-resistant strain belonging to ST15 was identified using MLST analysis. The alleles for the 7 housekeeping genes *atpD*, *gapA*, *guaA*, *mutM*, *nuoD*, *ppsA*, and *recA* were 10-29-21-21-32-32-10, respectively, with ST15 matching the strain found in China, India, and southern Poland [54, 60–62]. A study conducted in the South of Poland that focused on typing isolates of nonfermenting Gram-negative bacteria in blood infections revealed that one of the isolates was ST15. Additionally, seven other isolates had new STs [60]. Similarly in 2019, a study conducted in India identified a single isolate as being of sequence type 15 (ST15) [62]. Zamboni et al. revealed that the clinical and environmental strains of *S. maltophilia* were divided into five ST groups: ST304, ST239, ST24, ST150, and ST290 [16]. Consistent with prior research, various sequence types were ascertained for *S. maltophilia* isolates, encompassing STs 5, 4, 26, 31, 8, 14, 29, 39, 77, 78, 92, 93, 94, 122, and 152 [63, 64]. The widespread identification of ST15 across different geographic regions implies its global prevalence, potentially influencing *S. maltophilia*'s global epidemiology, and it provides a point of comparison for new research. The presence of ST15 in trimethoprim-sulfamethoxazole-resistant strains emphasizes the association between this sequence type and antibiotic resistance, although more research would be needed to confirm this. Overall, our study adds to the expanding understanding of *S. maltophilia's* genetic diversity and antibiotic resistance. This insight is vital for shaping treatment approaches and public health measures. Further research is needed to fully understand the implications of these findings and to continue monitoring the evolution and spread of *S. maltophilia*. Uncertainty about effective management strategies for *S. maltophilia* infections stems from the limited availability of treatment options, supported by both in vitro and clinical evidence. In addition, distinguishing between colonization and invasive infections caused by *S. maltophilia* can be a significant challenge. Based on promising in vitro activity and positive clinical outcomes, TMP-SMX is generally considered the preferred therapeutic option for the treatment of *S. maltophilia* infections.

## 5. Conclusion

Our study highlights the critical role of microbiology laboratories in the identification and understanding of antibiotic resistance and virulence factors in *S. maltophilia*. The high percentage of antibiotic resistance mechanisms among *S. maltophilia* strains isolated from hospitalized patients emphasizes the enhancement of laboratory expertise for accurate diagnosis and effective prevention of resistant pathogen transmission. The development of reliable assays for the detection of latent resistance is necessary, as is the importance of careful prescribing practices supported by antibiogram testing to ensure appropriate treatment strategies.

Our findings reveal a concerning rise in resistance to SXT, particularly worrisome given that *S. maltophilia* is already resistant to numerous antibiotics, leaving SXT as one of the few viable treatment options. Even minimal resistance levels pose challenges, as they limit therapeutic choices and complicate infection management. While current resistance reports remain low, the gradual increase warrants close monitoring and proactive intervention strategies to mitigate the potential escalation of resistance and preserve SXT's efficacy. Additionally, the identification of multiple virulence genes contributing to *S. maltophilia* highlights the importance of addressing both antibiotic resistance and virulence factors in the control of this opportunistic pathogen. These insights offer a way for future interventions and surveillance strategies, underscoring the need for a comprehensive approach that includes effective laboratory expertise, development of reliable assays, cautious prescribing practices, improved infection control measures, and a focus on both antibiotic resistance and virulence factors. This approach is vital for the effective management of *S. maltophilia*, which leads to enhance patient outcomes and reduce global mortality rates.

## Figures and Tables

**Table 1:** Oligonucleotide primers used in this study.

Gene	Primer sequences 5“-3”	Amplified fragment (bp)	Reference
*Primers for amplification of resistance and virulence factor genes*			
*16srRNA*	F: AGTTCGCATCGTTTAGGG	762	[30]
	R: ACGGCAGCACAGAAGAGC		
*bla*_*L*1_	F: ATCTACGGCTGATCCTGCTC	373	[24]
	R: GGATAGCGTGCGTTGTACTG		
*bla*_*L*2_	F: CGATTCCTGCAGTTCAGT	905	[21]
	R: CGGTTACCTCATCCGATC		
*stmPr1*	F: TGAAAGCAAATGCGCCGTT	850	[16]
	R: GTGATGGCGTCGGTGATGTC		
*stmPr2*	F: GCCGATTCCGGCATTCACACC	1764	[13]
	R: GGTCAGGCCCGAGAAGGTGCT		
*virB*	F: GCATCATGCAGAACGAGCTG	1075	[16]
	R: GACGGCTCGTACTTCTGCAC		
*hlyIII*	F: CGTCCATTGCTTCGATCCGTG	605	
	R: GACGAAGTGGCAGACGCTG		
*pilU*	F: CGACCACCATCGATTTCACTTCG	778	
	R: GACAGGTCCATCAGCAGCTG		
*papD*	F: CACGCGAGTGATCTATCCGG	579	
	R: GTGATGAAGCGCACCTGGTC		
*fliC*	F: CGATCTCCGAGCGCTTCG	296	
	R: GAACAGCTGGCTGGAGAACG		

*Primers used for MLST*			
*atpD*	F: ATGAGTCAGGGCAAGATCGTTC	854	[33]
	R: TCCTGCAGGACGCCCATTTC		
*gapA*	F: TGGCAATCAAGGTTGGTATCAAC	800	
	R: TTCGCTCTGTGCCTTCACTTC		
*gapA*	F: AGGAGCTTGAGAAATGGCAA	975	
	R: GAGTAGCCCCACTCGTTGTC		
*guaA*	F: AACGAAGAAAAGCGCTGGTA	700	
	R: ACGGATGGCGGTAGACCAT		
*mutM*	F: AACTGCCCGAAGTCGAAAC	614	
	R: GAGGATCTCCTTCACCGCATC		
*nuoD*	F: TTCGCAACTACACCATGAAC	514	
	R: CAGCGCGACTCCTTGTACTT		
*ppsA*	F: CAAGGCGATCCGCATGGTGTATTC	612	
	R: CCTTCGTAGATGAA(A/g)CCGGT(A/G)TC		
*recA*	F: ATGGACGAGAACAAGAAGCGC	783	
	R: GGTGATGACCTGCTTGAACGG		

atpD (H(+)-transporting two-sector ATPase)

gapA (NAD-dependent glyceraldehyde-3-phosphate dehydrogenase)

guaA (GMP synthase [glutamine-hydrolyzing])

mutM (DNA-formamidopyrimidine glycosylase)

nuoD (NADH dehydrogenase [ubiquinone])

ppsA (Pyruvate, water dikinase)

recA (RecA protein)

**Table 2:** The antimicrobial susceptibility profile exhibited by *S. maltophilia* isolates.

Antimicrobial agents	MIC (*μ*g/ml)			Disc diffusion number (%)		
	Range	MIC_50_	MIC_90_	Susceptible	Intermediate	Resistant
Meropenem	—	—	—	2 (2.5%)	—	78 (97.5%)
Imipenem	—	—	—	—	—	80 (100%)
Minocycline	—	—	—	70 (87.5%)	10 (12.5%)	—
Ceftazidime	8_32	8	32	2 (2.5%)	6 (7.5%)	72 (90%)
Levofloxacin				80 (100%)	—	—
Trimethoprim-sulfamethoxazole	2.38–4.76	≤2.38	≤4.76	75 (93.75%)	3 (3.75%)	2 (2.5%)

**Table 3:** Prevalence of virulence genes among 80 *S. maltophilia* isolates.

Virulence genes, no. (%)						
*fliC*	*virB*	*papD*	*pilU*	*hlyIII*	*stmPr1*	*stmPr2*
78 (97.5%)	77 (96.25%)	58 (72.5%)	77 (96.2%)	76 (95%)	31 (38.75%)	80 (100%)

FliC: flagellum

virB gene: (TSS4)

PapD: colonization

pilU: (pili type 4)

HlyIII: hemolysin

StmPr1: serine proteases

StmPr2: serine proteases

**Table 4:** The number of alleles of and sequence type (ST) of TMP-SMX-resistant *S. maltophilia* in the present study.

	*atpD*	*gapA*	*guaA*	*mutM*	*nuoD*	*ppsA*	*recA*	*ST*
Strain 78	Allele 10	Allele 29	Allele 21	Allele 21	Allele 32	Allele 32	Allele 10	15

## Data Availability

The data used to support the findings of this study are included in the article.
